# Socioeconomic disparity and the risk of contracting COVID-19 in South Korea: an NHIS-COVID-19 database cohort study

**DOI:** 10.1186/s12889-021-10207-y

**Published:** 2021-01-15

**Authors:** Tak Kyu Oh, Jae-Wook Choi, In-Ae Song

**Affiliations:** 1 Department of Anesthesiology and Pain Medicine, Seoul National University Bundang Hospital, Gumi-ro 173 Beon-gil, Bundang-gu, Seongnam, 13620 South Korea; 2 Department of Preventive Medicine, College of Medicine, Korea University, Seoul, South Korea

**Keywords:** Social class, Income, Infections, COVID-19

## Abstract

**Background:**

The relationship between socioeconomic status and the risk of contracting coronavirus disease (COVID-19) remains controversial. We aimed to investigate whether socioeconomic status affected the risk of contracting COVID-19 in the South Korean population.

**Methods:**

The NHIS-COVID-19 database cohort was used in this population-based study. We collected the data of COVID-19 patients who were diagnosed between January 1, 2020 and June 4, 2020 and those of the control population. The income levels of all individuals as of February 2020 were extracted, and study participants were classified into four groups based on quartiles: Q1 (the lowest) to Q4 (the highest). Data were statistically analyzed using multivariable logistic regression modeling.

**Results:**

In total, 122,040 individuals—7669 and 114,371 individuals in the COVID-19 and control groups, respectively—were included in the final analysis. The multivariable logistic regression model showed that the Q1 group had a 1.19-fold higher risk of contracting COVID-19 than the Q4 group, whereas the Q2 and Q3 groups showed no significant differences. In the 20–39 years age group, compared with the Q4 group, the Q3 and Q2 groups showed 11 and 22% lower risks of contracting COVID-19, respectively. In the ≥60 years age group, compared with the Q4 group, the Q1, Q2, and Q3 groups showed a 1.39-, 1.29-, and 1.14-fold higher risks of COVID-19, respectively.

**Conclusions:**

Lower socioeconomic status was associated with a higher risk of contracting COVID-19 in South Korea. This association was more evident in the older population (age ≥ 60 years), whereas both lower and higher socioeconomic statuses were associated with higher risks of contracting COVID-19 in the young adult population (in the 20–39 year age group). Strategies for the prevention of COVID-19 should focus on individuals of lower socioeconomic status and on young adults of higher and lower socioeconomic status.

**Supplementary Information:**

The online version contains supplementary material available at 10.1186/s12889-021-10207-y.

## Background

Since the first report of 27 cases of pneumonia with unknown etiology in Wuhan City, Hubei, China [1], the coronavirus disease (COVID-19) has become a global pandemic. The World Health Organization declared that the outbreak of COVID-19 in China was a public health emergency of international concern on January 30, 2020 [2] and subsequently declared it a pandemic on March 11, 2020 [3]. As of December 6, 2020, 66,504,022 cases of COVID-19 and 1,528,373 COVID-19-related deaths have been reported globally [4]. Currently, there is no available vaccine for COVID-19, and the disease continues to represent a global public health crisis [5, 6].

From a public health perspective, socioeconomic disparities can lead to health inequality with regard to COVID-19 [7]. People with lower socioeconomic status have been segregated to overcrowded urban housing centers and workplaces, making physical distancing and self-isolation difficult and leading to increased risks of contracting and spreading COVID-19 [8]. For example, more COVID-19-related deaths were reported in African American and Hispanic people than in Caucasian people in the United States [9, 10]. From an economic perspective, a cohort study including 92 hospitals in the United States showed that specific insurance status was associated with in-hospital mortality. In this study, Medicare insurance status was associated with mortality independently from age [11]. However, no study has examined in detail the direct effect of annual income level of a country’s population on COVID-19 risk.

In South Korea, the annual income level of all individuals is registered in the National Health Insurance Service (NHIS) database to determine the national health insurance premiums. Thus, the effect of the annual income level on COVID-19 risk among the South Korean population can be examined. In addition, the Korean government pays all medical charges for patients who are diagnosed with COVID-19 to ensure that all patients can receive appropriate in-hospital treatment free of charge. The impact of annual income levels on in-hospital mortality among COVID-19 patients considering the financial coverage provided by the NHIS was not investigated.

Therefore, we aimed to investigate whether socioeconomic status affected the risk of contracting COVID-19 among the South Korean population. In addition, we examined the effect of socioeconomic status on in-hospital mortality among patients diagnosed with COVID-19.

## Methods

### Study design and ethical statement

This population-based observational study was conducted and reported according to the Reporting of Observational Studies in Epidemiology guidelines [12]. The study protocol was approved by the Institutional Review Board (X-2004-604-905) and the Health Insurance Review and Assessment Service (NHIS-2020-1-291). The requirement for informed consent was waived because the data analyses were performed retrospectively using anonymized data derived from the South Korean NHIS database.

### NHIS-COVID-19 cohort database and study population

The NHIS-COVID-19 cohort database was developed for medical research purposes in cooperation between the NHIS and Korea Centers for Disease Control and Prevention (KCDC). The KCDC provides data on patients diagnosed with COVID-19 from January 1, 2020 to June 4, 2020, such as COVID-19 confirmation date, treatment results, and demographic information. In addition, the data of COVID-19 patients who are receiving ongoing in-hospital treatment are not included in this database because treatment results are not yet available. Using the data on COVID-19 patients, the NHIS extracts the control population using stratification methods regarding age, sex, and place of residence as of February 2020. The NHIS-COVID-19 cohort database contains disease diagnoses according to the International Classification of Diseases (ICD)-10 codes and prescription information concerning drugs and/or procedures from 2015 to 2020. For this study, an independent medical record technician at the NHIS center unaffiliated to the study extracted the data on June 26, 2020. In the analysis, we included individuals 20 years old or older because in the NHIS-COVID-19 cohort database, the NHIS provided information on age groups considering age as a categorical variable (20–29, 30–39, 40–49, 50–59, 60–69, 70–79, and ≥ 80 years) in conformance with the anonymized patient information in the database. In addition, individuals with an incomplete medical record were excluded; however, if an individual had missing data for only annual income levels due to the lack of information in the NHIS database, he/she was included in the analysis in the “unknown group” to avoid bias because sometimes, the annual income levels of military soldiers and individuals without health insurance coverage were not registered in the NHIS database.

### Annual income level

All individuals in South Korea are registered in the NHIS [13] and divided into two groups: employee insured and self-employed insured. The insurance premium for employee insured individuals is determined according to income, whereas that for self-employed insured individuals is determined according to income, property, living standards, and rate of participation in economic activities. South Koreans pay a fixed rate for health insurance premiums based on their income, with approximately 67% of their medical expenses being subsidized by the government [13]. However, those who cannot afford insurance premiums or have difficulty in financially supporting themselves are included in the Medical Aid Program. In this program, the government covers almost all medical expenses to reduce the financial burden of medical costs. For this study, we obtained data on insurance premiums and used it to derive the annual income level of all participants as of February 2020. The NHIS provided information on income levels to researchers based on 20 groups created according to 5% intervals. Therefore, for analysis, we employed two methods: First, the annual income levels were classified into 20 groups according to the 5% intervals (Group 1: 0–5% [lowest] to Group 20: 95–100% [highest]) to examine the linear trends for the risk of contracting COVID-19 and risk of mortality among COVID-19 patients according to the annual income level. Participants of the Medical Aid Program (constituting approximately 2.5% of the study population) were included in the Group 1 (0–5%). Second, the annual income level was classified into four groups using quartile ratios (from Q1, the lowest, to Q4, the highest). In addition, we used the data from 2015 to 2020 to calculate the 6-year average income level and included it in the analysis. Importantly, in South Korea, all COVID-19 patients were treated in the hospital free of charge regardless of their income level.

### Disability of individuals

In South Korea, all individuals with disability are registered in the NHIS database to receive various benefits. We extracted the data on registered disabilities of all participants as of February 2020. The degrees of disabilities were divided into two groups according to severity criteria: severe disability and mild to moderate disability. The types of disabilities were divided into five groups: physical disability, brain lesion disability, visual disturbance, hearing disability, and other disabilities.

### Endpoints

The primary endpoint of this study was development of COVID-19 in the NHIS-COVID-19 database cohort. We evaluated this endpoint from January 1, 2020 to June 4, 2020. The secondary endpoint of this study was in-hospital mortality among patients diagnosed with COVID-19.

### Other measurements as confounders

The data extracted as confounders included demographic characteristics (age and sex), place of residence (Seoul, Gyeonggi-do, Daegu, Gyeongsangbuk-do, and other areas), and the Charlson comorbidity index (CCI), which was calculated based on the registered ICD-10 diagnostic codes (Additional file 1) from January 1, 2015 to December 31, 2019. Age was divided into seven groups: 20–29, 30–39, 40–49, 50–59, 60–69, 70–79, and ≥ 80 years. Data on the variables of age, sex, and indicators of underlying diseases, such as the CCI, were collected, and these variables were considered as confounders because they were reported to be associated with the risk of contracting COVID-19 and the risk of COVID-19 mortality [14]. The place of residence was recorded as an important confounder because there were major outbreaks of COVID-19 in Daegu and Gyeongsangbuk-do until June 4, 2020 [15], and this may have affected the results.

### Statistical analysis

The characteristics of COVID-19 patients and those in the control group were compared using the Student’s t-test for continuous variables (CCI [because the CCI followed a normal distribution]) and the chi-square test for categorical variables (all other variables). First, we investigated the relationship between the risk of contracting COVID-19 and income level in 2020 using restricted cubic splines (RCS). Second, we constructed a multivariable logistic regression model to analyze the development of COVID-19 among the NHIS-COVID-19 database cohort. All confounders were included in the model for multivariable adjustment. Income level in 2020 was included as two types of independent variables to avoid multicollinearity in the model: 1) categorical variables using quartile ratios and 2) continuous variables using 5%-increase intervals to examine if there were linear trends for the risk of contracting COVID-19 and risk of mortality among COVID-19 patients according to the annual income level. In the sensitivity analysis, the 6-year average income level was included in a separate multivariable model to investigate whether the average income level for 6 years (2015–2020), was associated with risk of COVID-19 and mortality among COVID-19 patients. The grade and type of disability were also included in the separate multivariable model to avoid multicollinearity in the model. Additionally, the CCI and diseases used to calculate the index were included in the separate model to avoid multicollinearity. Third, we performed subgroup analyses according to age. All participants were classified into three subgroups according to age (20–39, 40–59, and ≥ 60 years groups). Therefore, multivariable model 1 included the income level in 2020 as a categorical variable using quartile ratios; model 2 included the income level in 2020 as a continuous variable (per 5% decrease); model 3 included the average income level for 6 years (2015–2020) for sensitivity analysis. In addition, the grade of disability, and CCI were included in multivariable model 1, whereas the type of disability and specific underlying diseases, which were used to calculate the CCI, were included in multivariable model 2. The subgroup analyses were performed using the same methods as those described for the main analyses. Finally, we performed multivariable logistic regression analysis for in-hospital mortality among patients diagnosed with COVID-19 to investigate whether income level in 2020 affected their mortality after the South Korean government subsidized all hospital charges for COVID-19 patients during the period of the study.

The results of the logistic regression models are presented as odds ratio (OR) with 95% confidence intervals (CIs). Using a variance inflation factor < 2.0, we confirmed that multicollinearity occurred in none of the multivariable models. Additionally, we performed the Hosmer–Lemeshow test to examine the goodness-of-fit of the multivariable models for the entire cohort. A receiver operating characteristic (ROC) analysis was performed for validation purposes in this study. The R (version 3.6.3; R Foundation for Statistical Computing, Vienna, Austria) was used for all analyses. *P* < 0.05 was considered statistically significant.

## Results

### Study population

**Fig. 1:**
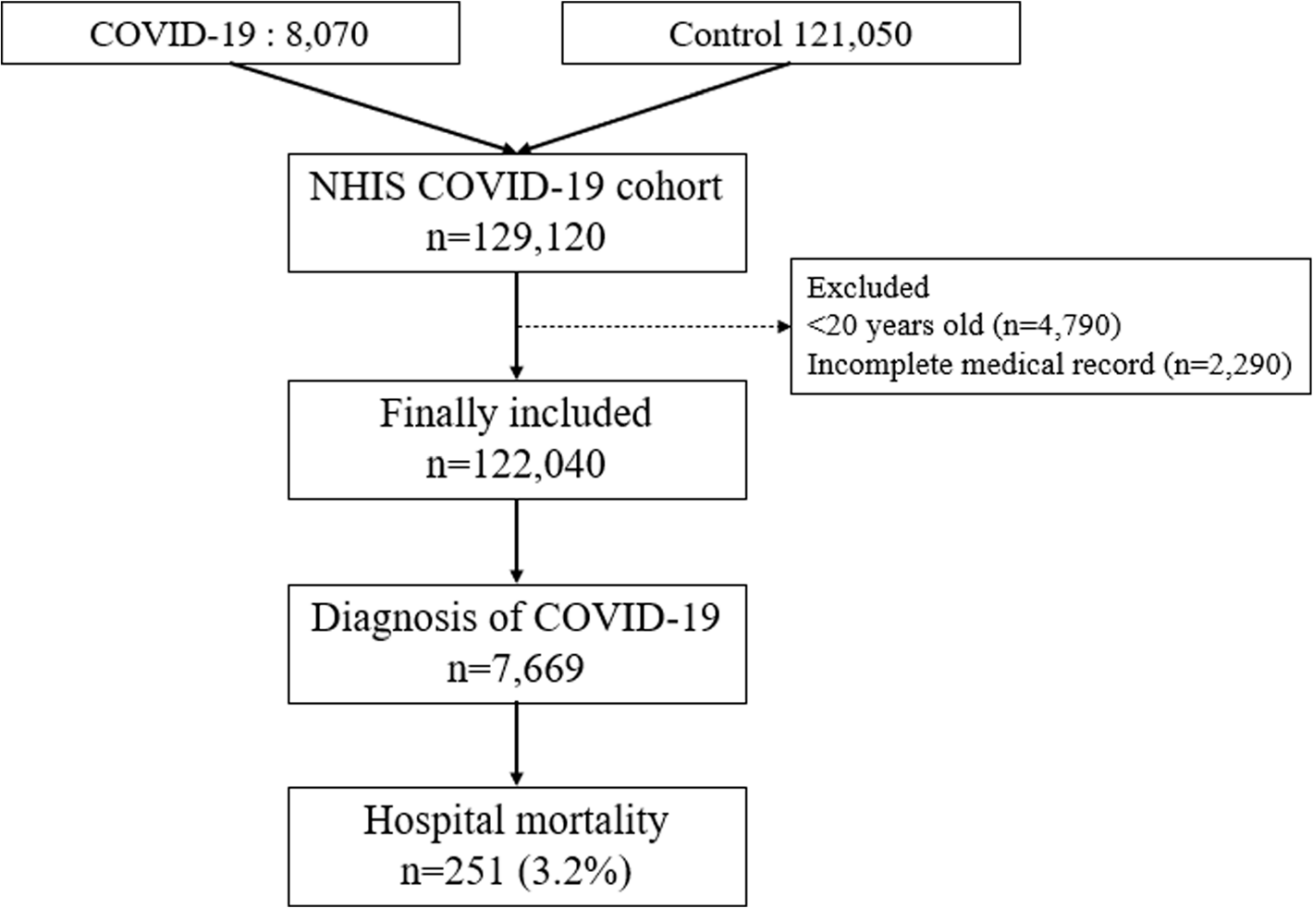
Flowchart of the participant selection process

As of June 4, 2020, the NHIS-COVID-19 database cohort comprised 8070 COVID-19 patients and 121,050 individuals in the control population. Among them, 4790 individuals younger than 20 years and 2290 with incomplete medical records were excluded from the analysis. Thus, 122,040 individuals were included in the final analysis, and 7669 individuals were diagnosed with COVID-19 during the study period. Of these, 251 (3.2%) died due to COVID-19 during hospitalization (Fig. 1). Table 1 presents the results of comparison of characteristic between COVID-19 patients and control population in South Korea.

**Table 1:** Comparison of characteristic between COVID-19 patients and control group in South Korea in 2020

Variable	Control	COVID-19	*P*-value
	*n* = 114,371	*n* = 7669	
Age			0.998
20–29	30,306 (26.5)	2051 (26.7)	
30–39	12,238 (10.7)	828 (10.8)	
40–49	15,391 (13.5)	1029 (13.4)	
50–59	23,282 (20.4)	1558 (20.3)	
60–69	17,869 (15.6)	1186 (15.5)	
70–79	9223 (8.1)	613 (8.0)	
≥ 80	6062 (5.3)	404 (5.3)	
Sex, male	45,094 (39.4)	3024 (39.4)	1.00
Residence at Februrary, 2020			0.993
Seoul	7481 (6.5)	507 (6.6)	
Gyeonggi-do	74,931 (65.5)	5012 (65.4)	
Daegu	6320 (5.5)	418 (5.5)	
Gyeongsangbuk-do	13,816 (12.1)	932 (12.2)	
Other area	11,823 (10.3)	800 (10.4)	
Underlying disability	6566 (5.7)	608 (7.9)	< 0.001
Grade of disability			< 0.001
Severe	2221 (1.9)	291 (3.8)	
Mild to moderate	4345 (3.8)	317 (4.1)	
Type of disability			< 0.001
No disability	107,805 (94.3)	7061 (92.1)	
Physical disability	2854 (2.5)	214 (2.8)	
Brain lesion disability	649 (0.6)	72 (0.9)	
Visual disturbance	656 (0.6)	39 (0.5)	
Hearing disability	1185 (1.0)	102 (1.3)	
Other disability	1222 (1.1)	181 (2.4)	
Income level in 2020 (Feb)			< 0.001
Q1 (Lowest)	29,272 (25.6)	2428 (31.7)	
Q2	23,190 (20.3)	1441 (18.8)	
Q3	25,761 (22.5)	1554 (20.3)	
Q4 (Highest)	34,177 (29.9)	2130 (27.8)	
unknown	1971 (1.7)	116 (1.5)	
Average Income level (2015–2020)			< 0.001
Q1 (Lowest)	13,363 (11.7)	961 (12.5)	
Q2	29,932 (26.2)	2016 (26.3)	
Q3	30,275 (26.5)	1814 (23.7)	
Q4 (Highest)	30,221 (26.4)	1879 (24.5)	
unknown	10,580 (9.3)	999 (13.0)	
Charlson comorbidity index 2020	1.7 (2.8)	3.3 (3.5)	< 0.001
Peripheral vascular disease	6311 (5.5)	454 (5.9)	0.143
Renal disease	1138 (1.0)	132 (1.7)	< 0.001
Rheumatic disease	1936 (1.7)	232 (3.0)	< 0.001
Dementia	3233 (2.8)	418 (5.5)	< 0.001
Peptic ulcer disease	8417 (7.4)	1014 (13.2)	< 0.001
DM without chronic complication	11,511 (10.1)	1578 (20.6)	< 0.001
Hemiplegia or paraplegia	434 (0.4)	104 (1.4)	< 0.001
Moderate or severe liver disease	121 (0.1)	15 (0.2)	0.035
Mild liver disease	11,296 (9.9)	1745 (22.8)	< 0.001
Chronic pulmonary disease	11,094 (9.7)	2454 (32.0)	< 0.001
DM with chronic complication	3656 (3.2)	303 (4.0)	< 0.001
Cerebrovascular disease	4926 (4.3)	478 (6.2)	< 0.001
Congestive heart failure	2792 (2.4)	668 (8.7)	< 0.001
Myocardial infarction	755 (0.7)	344 (4.5)	< 0.001
Metastatic solid tumor	18,847 (16.5)	2341 (30.5)	< 0.001
Malignancy	3524 (3.1)	332 (4.3)	< 0.001
AIDS/HIV	22 (0.0)	10 (0.1)	< 0.001

Presented as mean with standard deviation or number with percentage

*DM* diabetes mellitus, *AIDS* acquired immune deficiency syndrome, *HIV* Human Immunodeficiency Virus

### COVID-19 risk

The RCS in Additional File 2 shows that the log odds of severe acute respiratory syndrome coronavirus-2 (SARS-CoV-2) infection risk increased in those with an income level above the median value in 2020. In the 20–39-year-old subgroup, the RCS in Additional File 3 shows a U-shape between income level in 2020 and the log odds of SARS-CoV-2 infection risk, suggesting that both the highest and lowest income levels were associated with higher risks of COVID-19. In the 40–59-year-old subgroup, the RCS in Additional File 4 shows a similar pattern for all individuals as shown in Additional File 2. However, in the ≥60-year-old subgroup, the RCS in Additional File 5 shows that the log odds of SARS-CoV-2 infection risk gradually increased as the income level in 2020 decreased, suggesting that income level and COVID-19 risk were inversely related.

Table 2 presents the multivariable logistic regression models for the diagnosis of COVID-19 in South Korea. Regarding income levels in 2020, the Q1 group had a 1.19-fold higher COVID-19 risk than the Q4 group (OR: 1.19, 95% CI; 1.12–1.27; *P* < 0.001; model 1), whereas the Q3 (*P* = 0.06) and Q2 (*P* = 0.26) groups showed no significant differences. An income level decrease of 5% was associated with an increase of 1% in COVID-19 risk (OR: 1.01, 95% CI: 1.01–1.02; *P* < 0.001; model 2). In the sensitivity analysis, the Q1 group had a 1.10-fold higher COVID-19 risk than the Q4 group (OR: 1.10, 95% CI: 1.01–1.19; *P* = 0.028 model 3). In addition, individuals with severe disabilities had a 1.45-fold higher COVID-19 risk than individuals without disabilities (OR: 1.45, 95% CI: 1.26–1.67; *P* < 0.001; model 1), whereas mild to moderate disabilities were not significantly associated with a higher risk for contracting COVID-19 (*P* = 0.088). Among the types of disabilities, brain lesion disability was specifically associated with a 1.32-fold higher COVID-19 risk (OR: 1.32, 95% CI: 1.02–1.70; *P* = 0.033; model 2). Hosmer–Lemeshow statistics showed appropriate goodness-of-fit in the three models (all *P* > 0.05). The area under the curve of the multivariable models in the ROC analyses was 0.81 (95% CI: 0.80–0.81).

**Table 2:** Multivariable logistic regression models for diagnosis of COVID-19 in South Korea

Variable	Multivariable model	*P*-value
	OR (95% CI)	
Income level 2020 (Feb), model 1		
Q4 (Highest)	1	
Q3	0.94 (0.87, 1.01)	0.062
Q2	0.96 (0.89, 1.03)	0.258
Q1 (Lowest)	1.19 (1.12, 1.27)	< 0.001
unknown	0.92 (0.76, 1.12)	0.425
Income level 2020 (Feb), per decrease 5%, model 2	1.01 (1.01, 1.02)	< 0.001
Average Income level (2015–2020), model 3		
Q4 (Highest)	1	
Q3	0.93 (0.87, 1.05)	0.062
Q2	1.03 (0.96, 1.10)	0.361
Q1 (Lowest)	1.10 (1.01, 1.19)	0.028
unknown	1.25 (1.15, 1.37)	< 0.001
Age, year		
20–29	1	
30–39	0.91 (0.84, 0.99)	0.321
40–49	0.81 (0.75, 0.88)	< 0.001
50–59	0.69 (0.64, 0.74)	< 0.001
60–69	0.51 (0.47, 0.55)	< 0.001
70–79	0.36 (0.32, 0.40)	< 0.001
≥ 80	0.28 (0.24, 0.32)	< 0.001
Sex, male	1.02 (0.97, 1.07)	0.493
Residence at Februrary, 2020		
Seoul	1	
Gyeonggi-do	0.92 (0.83 1.01)	0.090
Daegu	0.97 (0.85 1.12)	0.693
Gyeongsangbuk-do	0.90 (0.80 1.02)	0.092
Other area	0.89 (0.79 1.01)	0.063
Grade of disability (model 1)		
No disability	1	
Severe	1.45 (1.26 1.67)	< 0.001
Mild to moderate	0.89 (0.78 1.02)	0.088
Type of disability (model 2)		
No disability	1	
Physical disability	1.01 (0.87, 1.17)	0.872
Brain lesion disability	1.32 (1.02, 1.70)	0.033
Visual disturbance	0.83 (0.59, 1.15)	0.254
Hearing disability	1.20 (0.97, 1.48)	0.099
Other disability	1.82 (1.55, 2.15)	< 0.001
Charlson comorbidity index 2020, 1 point (model 1)	1.19 (1.18, 1.20)	< 0.001
Peripheral vascular disease	0.69 0.62 0.77	< 0.001
Renal disease	0.84 0.68 1.03	0.093
Rheumatic disease	1.08 0.93 1.25	0.330
Dementia	1.97 1.72 2.25	< 0.001
Peptic ulcer disease	1.26 1.17 1.36	< 0.001
DM without chronic complication	1.69 1.57 1.82	< 0.001
Hemiplegia or paraplegia	2.36 1.83 3.05	< 0.001
Moderate or severe liver disease	0.62 0.34 1.14	0.123
Mild liver disease	1.90 1.77 2.03	< 0.001
Chronic pulmonary disease	3.62 3.42 3.83	< 0.001
DM with chronic complication	0.77 0.67 0.88	< 0.001
Cerebrovascular disease	0.94 0.84 1.06	0.328
Congestive heart failure	2.40 2.16 2.67	< 0.001
Myocardial infarction	4.64 4.00 5.38	< 0.001
Metastatic solid tumor	1.70 1.61 1.80	< 0.001
Malignancy	0.89 0.79 1.01	0.084
AIDS/HIV	3.39 1.46 7.87	0.005

AUC of multivariable model 1,2, and 3: 0.81 (95% CI: 0.80, 0.81)

Multivariable model 1 included the income level in 2020 as a categorical variable using quartile ratios; model 2 included the income level in 2020 as a continuous variable (per 5% decrease); model 3 included the average income level for 6 years (2015–2020) for sensitivity analysis. In addition, the grade of disability, and CCI were included in multivariable model 1, whereas the type of disability and specific underlying diseases, which were used to calculate the CCI, were included in multivariable model 2

*OR* odds ratio, *CI* confidence interval, *DM* diabetes mellitus, *AIDS* acquired immune deficiency syndrome, *HIV* Human Immunodeficiency Virus, *AUC* area under curve

Table 3 presents the results of subgroup analyses according to age. Regarding income levels in 2020, in the 20–39-year-old group, the Q3 and Q2 groups had 11% (OR: 0.89, 95% CI: 0.81–0.97; *P* = 0.001) and 22% (OR: 0.78, 95% CI: 0.90–1.11; *P* < 0.001) lower risks of COVID-19, respectively, than the Q4 group, whereas the Q1 group showed no significant difference (*P* = 0.99). In the 40–59-year-old group, the Q1 group had a 1.23-fold (OR: 1.23, 95% CI: 1.10–1.37; *P* < 0.001) higher COVID-19 risk than the Q4 group, whereas the Q2 (*P* = 0.49) and Q3 (*P* = 0.11) groups showed no significant differences. In the ≥60-year-old group, the Q1, Q2, and Q3 groups showed 1.39-fold (OR: 1.39, 95% CI: 1.24–1.56; *P* < 0.001), 1.29-fold (OR: 1.29, 95% CI: 1.12–1.48; *P* < 0.001), and 1.14-fold (OR: 1.14, 95% CI: 1.01–1.30; *P* = 0.05) higher risks of COVID-19 than the Q4 group.

**Table 3:** Subgroup analyses according to age

Variable	Multivariable model	*P*-value
	OR (95% CI)	
Age 20–39 (*n* = 45,423)		
Income level 2020 (Feb), per increase 5% (model 1)	1.00 (0.99, 1.01)	0.809
Income level 2020 (Feb) (model 2)		
Q4 (Highest)	1	
Q3	0.89 (0.81, 0.97)	0.001
Q2	0.78 (0.69, 0.87)	< 0.001
Q1 (Lowest)	1.00 (0.90, 1.11)	0.995
unknown	0.68 (0.50, 0.93)	0.016
Age 40–59 (*n* = 41,260)		
Income level 2020 (Feb), per increase 5% (model 1)	1.02 (1.01, 1.02)	< 0.001
Income level 2020 (Feb) (model 2)		
Q4 (Highest)	1	
Q3	0.90 (0.80, 1.02)	0.106
Q2	0.96 (0.84, 1.08)	0.487
Q1 (Lowest)	1.23 (1.10, 1.37)	< 0.001
unknown	1.22 (0.87, 1.73)	0.250
Age ≥ 60 (*n* = 35,357)		
Income level 2020 (Feb), per increase 5% (model 1)	1.02 (1.01, 1.03)	< 0.001
Income level 2020 (Feb) (model 2)		
Q4 (Highest)	1	
Q3	1.14 (1.01, 1.30)	0.048
Q2	1.29 (1.12, 1.48)	< 0.001
Q1 (Lowest)	1.39 (1.24, 1.56)	< 0.001
unknown	0.95 (0.64, 1.43)	0.815

*OR* odds ratio, *CI* confidence interval

### Hospital mortality in COVID-19 patients

Table 4 presents the results of multivariable logistic regression models for in-hospital mortality in COVID-19 patients in South Korea. Regarding income levels in 2020, the Q1 (*P* = 0.30), Q2 (*P* = 0.53), and Q3 (*P* = 0.32) groups were not associated with in-hospital mortality among COVID-19 patients compared with that of the Q4 group. In addition, individuals with severe disabilities had a 2.82-fold higher hospital mortality risk than individuals without disabilities (OR: 2.82, 95% CI: 1.68–4.73; *P* < 0.001), whereas individuals with mild to moderate disabilities did not have a significantly higher mortality risk (*P* = 0.557).

**Table 4:** Multivariable logistic regression models for hospital mortality in COVID-19 patients in South Korea (*n* = 7669, death = 233, 3.0%)

Variable	Multivariable model	*P*-value
	OR (95% CI)	
Income level 2020 (Feb), per increase 5% (model 1)	1.01 (0.99, 1.03)	0.472
Income level 2020 (Feb) (model 2)		
Q4 (Highest)	1	
Q3	1.25 (0.80, 1.94)	0.322
Q2	1.18 (0.71, 1.96)	0.526
Q1 (Lowest)	1.23 (0.83, 1.80)	0.300
unknown	0.68 (0.17, 2.71)	0.586
AGE, 10 year increase	3.23 (2.70, 3.86)	< 0.001
Sex, male	2.48 (1.81,3.41)	< 0.001
Residence at Februrary, 2020		
Seoul	1	
Gyeonggi-do	2.86 (0.79, 10.36)	0.109
Daegu	3.39 (0.80, 14.33)	0.096
Gyeongsangbuk-do	2.49 (0.66, 9.33)	0.176
Other area	2.41 (0.60, 9.72)	0.216
Grade of disability		
No disability	1	
Severe	2.82 (1.68, 4.73)	< 0.001
Mild to moderate	0.87 (0.55, 1.38)	0.557
Charlson comorbidity index, 1 point (model 1)	1.27 (1.04, 1.55)	0.017
Peripheral vascular disease	1.13 (0.76, 1.69)	0.549
Renal disease	1.57 (0.93, 2.64)	0.093
Rheumatic disease	0.39 (0.18, 0.84)	0.016
Dementia	1.87 (1.27, 2.73)	0.001
Peptic ulcer disease	1.10 (0.76, 1.60)	0.620
DM without chronic complication	1.74 (1.13, 2.69)	0.013
Hemiplegia or paraplegia	1.59 (0.83, 3.07)	0.163
Moderate or severe liver disease	3.50 (0.87, 14.12)	0.078
Mild liver disease	0.78 (0.56, 1.09)	0.150
Chronic pulmonary disease	1.69 (1.23, 2.33)	0.001
DM with chronic complication	1.74 (1.13, 2.69)	0.013
Cerebrovascular disease	0.73 (0.48, 1.10)	0.131
Congestive heart failure	1.89 (1.35, 2.66)	< 0.001
Myocardial infarction	0.96 (0.56, 1.64)	0.879
Malignancy	1.02 (0.73, 1.42)	0.905
Metastatic solid tumor	1.96 (1.26, 3.05)	0.003
AIDS/HIV	1.19 (0.09, 15.64)	0.893

AUC of multivariable model 1 and 2: 0.83 (95% CI: 0.82, 0.83)

Multivariable model 1 included the income level in 2020 as a categorical variable using quartile ratios; model 2 included the income level in 2020 as a continuous variable (per 5% decrease); model 3 included the average income level for 6 years (2015–2020) for sensitivity analysis. In addition, the grade of disability, and CCI were included in multivariable model 1, whereas specific underlying diseases, which were used to calculate the CCI, were included in multivariable model 2

*OR* odds ratio, *CI* confidence interval, *DM* diabetes mellitus, *AIDS* acquired immune deficiency syndrome, *HIV* Human Immunodeficiency Virus, *AUC* area under curve

## Discussion

Using the NHIS-COVID-19 database cohort, we showed that lower socioeconomic status was associated with higher risk of contracting COVID-19 among the South Korean population. Interestingly, this trend was the most evident in the population 60 years old or older, whereas both lower and higher socioeconomic status were associated with higher contracting COVID-19 in the population 20–39 years old. It suggests that preventive strategies for COVID-19 should focus on individuals of lower socioeconomic status in general and of both higher and lower socioeconomic status in young adults. Additionally, considering that all COVID-19 treatment in South Korea was free of charge, socioeconomic status was not associated with in-hospital mortality among COVID-19 patients, suggesting that financial coverage is an important factor for better prognosis of COVID-19 patients regardless of socioeconomic status. Additionally, our study showed that having severe disabilities was associated with higher risks of COVID-19 in the general population and with higher in-hospital mortality among COVID-19 patients, suggesting that individuals with severe disabilities require special considerations regarding prevention and treatment strategies for COVID-19.

The higher risk of COVID-19 in the population with lower socioeconomic status is an important finding in developing or low-income countries. In general, the medical and human resources that provide community adaptive systems against contracting COVID-19 during the pandemic are lacking in developing or low-income countries, as reported in Vietnam and Ghana [16, 17]. A previous study reported that the social insurance system in low- and middle-income countries typically covered a much smaller share of medical costs than that covered in high-income countries [18]. Therefore, in developing or low-income countries, people with lower socioeconomic status might face difficulty in receiving social protection against COVID-19, compared to people with a higher socioeconomic status. In this study, the results suggested that the implementation of an appropriate medical delivery system and adequate resource distribution in people with lower socioeconomic status can be critical issues in developing or low-income countries.

Many studies focused on the impact of disparity race or ethnicity disparities on the risk and mortality of COVID-19, [8, 9, 19–21] but information regarding the relationship between income level and COVID-19 risk was insufficient. The Centers for Disease Control and Prevention in the United States reported that annual household incomes below $25,000 were associated with higher risks of developing severe COVID-19 in a nationally representative survey [20]. However, they did not evaluate the effect of annual income levels of individuals in detail. A study in the United Kingdom reported that lower-income individuals are more likely to have underlying comorbidities that make them vulnerable to COVID-19, such as asthma, congestive heart failure, coronary heart disease, cancer, and hypertension [22]. However, they did not evaluate the direct relationship between income level and COVID-19 risk. Considering the limitation of previous studies, [20, 22] we showed the direct relationship between socioeconomic status and COVID-19 risk in the South Korean population.

Our study showed that besides those of lower socioeconomic status, young adults (20–39 years) of higher socioeconomic status have higher COVID-19 risks. In Japan, COVID-19 was transmitted in music clubs owing to asymptomatic carriers [23]. Although there is not enough information regarding this issue, young adults of higher socioeconomic status might engage in social meetings or visits in music clubs, making physical distancing difficult. Furthermore, the fact that young college students may have their socioeconomic status determined by their parents’ may be a source of bias in this relationship. Nonetheless, further studies are necessary to elucidate this issue.

The results regarding the relationship between in-hospital mortality and socioeconomic status are important for public health because the South Korean government subsidizes hospital charges for all COVID-19 patients. COVID-19 significantly increases the need for medical supplies, [24] and a single symptomatic COVID-19 patient could incur a median direct medical cost of $3045 during the course of the infection alone [25]. Therefore, the financial burden of COVID-19 treatment may be a significant issue among COVID-19 patients. In the United States, COVID-19 patients with Medicare insurance had higher in-hospital mortality rates than patients with commercial insurance [11]. Conversely, our study suggests that in-hospital mortality rates were not influenced by the socioeconomic status of COVID-19 patients owing to the total financial coverage system in South Korea.

Our study showed that individuals with severe disabilities had higher COVID-19 risks and in-hospital mortality after COVID-19 diagnosis. Individuals with severe disabilities have comorbidities that may be related with increased COVID-19 risks. Furthermore, disability represents a significant health issue among elderly people [26]. As confirmed by our study, the mortality of elderly COVID-19 patients is higher than that of young and middle-aged patients [27]. Therefore, the results regarding severe disability may be influenced by older age and underlying comorbidities.

Our study had several limitations. First, important variables—such as body mass index or smoking history—were not included in the analysis because they are not registered in the NHIS database. Second, we used the ICD-10 codes registered in the NHIS database to calculate the CCI, but some of the codes might not reflect the actual underlying diseases. Furthermore, we did not evaluate the impact of psychiatric illnesses on the risk of contracting COVID-19 and risk of COVID-19-related mortality in this study. Given that COVID-19 is associated with mental health in the general population [28, 29], the non-inclusion of this relevant factor might be considered an important limitation of this study. Finally, the multivariable adjustment only controls for known confounders, and residual or unmeasured confounders may still be present in this study.

## Conclusions

Our study showed that, in general, lower socioeconomic status was associated with higher risks of contracting COVID-19 in South Korea. This association was more evident in the older population, whereas both lower and higher socioeconomic statuses were associated with higher risks of contracting COVID-19 in young adults. Preventive strategies for COVID-19 should focus on individuals of lower socioeconomic status and on young adults of higher and lower socioeconomic status. Additionally, because of the full coverage of hospital charges for South Korean COVID-19 patients, socioeconomic status was not associated with in-hospital mortality in these patients.

## Supplementary Information

**Additional file 1.** The ICD-10 codes for comorbidities used to compute the Charlson comorbidity index.

**Additional file 2.** Restricted cubic spline for SARS-CoV-2 infection risk in the total cohort according to income level.

**Additional file 3.** Restricted cubic spline for SARS-CoV-2 infection risk in the 20–39-year-old subgroup according to income level.

**Additional file 4.** Restricted cubic spline for SARS-CoV-2 infection risk in the 40–59-year-old subgroup according to income level.

**Additional file 5.** Restricted cubic spline for SARS-CoV-2 infection risk in the ≥60-year-old subgroup according to income level.

## Data Availability

The data that support the findings of this study areavailable from National Health Insurance System, but restrictions apply to the availability of these data, which were used under licence for the current study and so are not publicly available. Data are, however, available from the authors upon reasonable request and with permission from the National Health Insurance System (https://nhiss.nhis.or.kr/bd/ab/bdaba000eng.do).

## Abbreviations

COVID-19: Coronavirus disease 2019
NHIS: National Health Insurance Service
KCDC: Korea Centers for Disease Control and Prevention
ICD: International Classification of Diseases
RCS: Restricted cubic splines
OR: Odds ratio
CI: Confidence interval
ROC: Receiver operating characteristic
