# Supplementary figures and images for: Socioeconomic disparity and the risk of contracting COVID-19 in South Korea: an NHIS-COVID-19 database cohort study
Source: BMC Public Health. 2021 Jan 15;21:144. doi: 10.1186/s12889-021-10207-y (PMC7809637; doi:10.1186/s12889-021-10207-y)

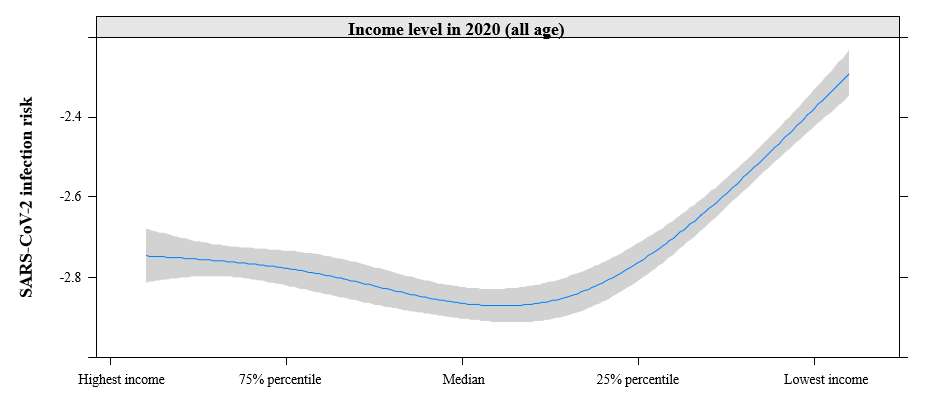

Supplement: Supplementary file 2 — Additional file 2. Restricted cubic spline for SARS-CoV-2 infection risk in the total cohort according to income level. [file 12889_2021_10207_MOESM2_ESM.tif]

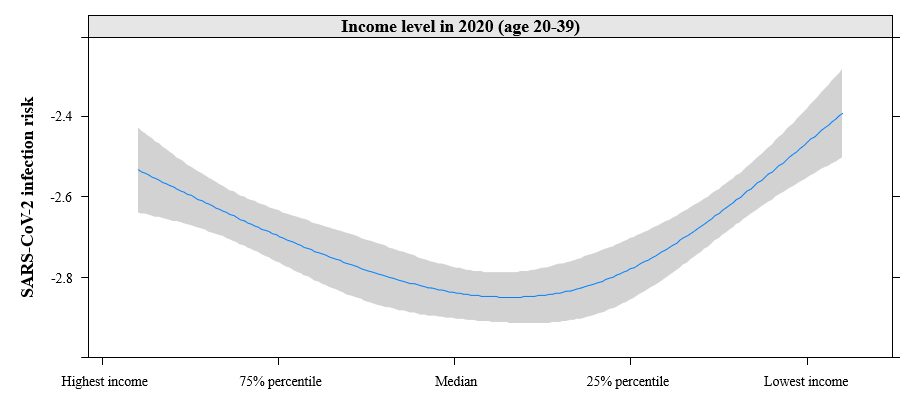

Supplement: Supplementary file 3 — Additional file 3. Restricted cubic spline for SARS-CoV-2 infection risk in the 20–39-year-old subgroup according to income level. [file 12889_2021_10207_MOESM3_ESM.tif]

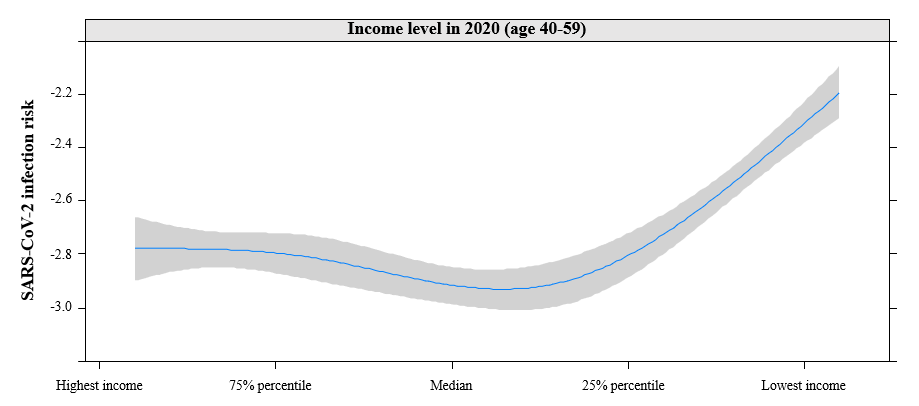

Supplement: Supplementary file 4 — Additional file 4. Restricted cubic spline for SARS-CoV-2 infection risk in the 40–59-year-old subgroup according to income level. [file 12889_2021_10207_MOESM4_ESM.tif]

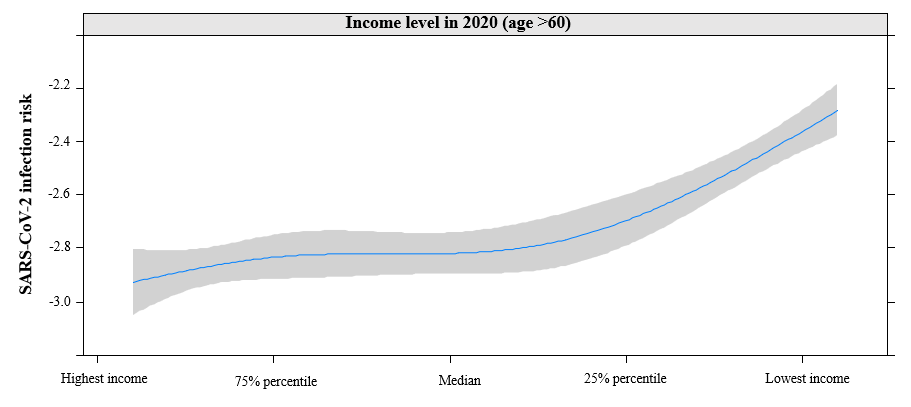

Supplement: Supplementary file 5 — Additional file 5. Restricted cubic spline for SARS-CoV-2 infection risk in the ≥60-year-old subgroup according to income level. [file 12889_2021_10207_MOESM5_ESM.tif]
